# Intra-thoracic desmoid tumour in a patient with a previous aortocoronary bypass

**DOI:** 10.1186/1477-7819-4-43

**Published:** 2006-07-10

**Authors:** Giuseppe Borzellino, Anna Maria Minicozzi, Francesco Giovinazzo, Giuseppe Faggian, Paolo Iuzzolino, Claudio Cordiano

**Affiliations:** 1 OCM Borgo Trento, Department of Surgery, Università di Verona, Ospedale Civile Maggiore Borgo Trento, 37126 Verona, Italy; 2 Department of Cardiac Surgery, Università di Verona, Ospedale Civile Maggiore Borgo Trento, 37126 Verona, Italy; 3 Division of Pathology, Università di Verona, Ospedale Civile Maggiore Borgo Trento, 37126 Verona, Italy

## Abstract

**Background:**

Intra-thoracic desmoid tumours with mediastinal invasion are very rare. Although rare they have to be taken into account in the differential diagnosis of a thoracic mass and therapeutic options have to be weighted since surgical treatment may require wide excision.

**Case presentation:**

A 48-year-old male diabetic, dyslipidaemic, former heavy smoker with psychiatric illness was operated by sternotomy for a triple aorto-coronary bypass 4 years before, presented with complains of recent onset such as constant and oppressive chest pain. At surgery a mass extending from the aortic arch into the entire anterior mediastinum and to most of the right pleural cavity was found. The mass was separated from sternal periosteum and vessels of aorto-coronary by pass were isolated starting from the aortic arch up to the pericardium. The histological examination revealed aggressive fibromatosis.

**Conclusion:**

Although technically demanding, radical surgical excision is actually the most indicated therapeutic approach for intra-thoracic desmoid tumours.

## Background

Desmoid tumours are soft-tissue neoplasms arising from fascial or musculo-aponeurotic structures [1]. They are characterized by the proliferation of fibroblasts with no cytological evidence of malignancy and are organized in the form of fasciae. They are considered benign because of their cytological features and because they don't give rise to metastases [2], but their biological behaviour is that of a locally aggressive tumour, relapse of which is not infrequent after surgical resection [3].

Intra-thoracic desmoid tumours with invasion of the mediastinum are however rare. We report here a case of an intra-thoracic desmoid tumour in a patient previously operated on by sternotomy for a triple aorto-coronary bypass.

## Case presentation

The patient was a 48-year-old male, diabetic, dyslipidaemic, former heavy smoker and suffering from paranoid schizophrenia, a triple aorto-coronary bypass was performed 4 years before by sternotomy. The patient was healthy till a few days before admission to the hospital, when he suffered constant oppressive chest pain.

**Figure 1:**
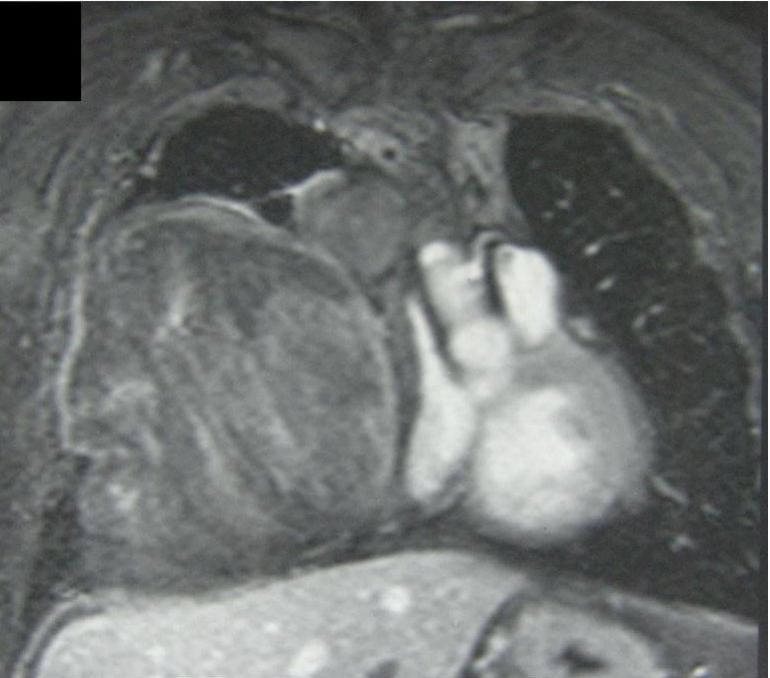
**MRI sagital scan showing mass extending into the mediastinum and right pleural cavity**. This scan shows clearly the anatomical relationship between the tumour and one of the vessels of the aorto-coronary by-pass.

**Figure 2:**
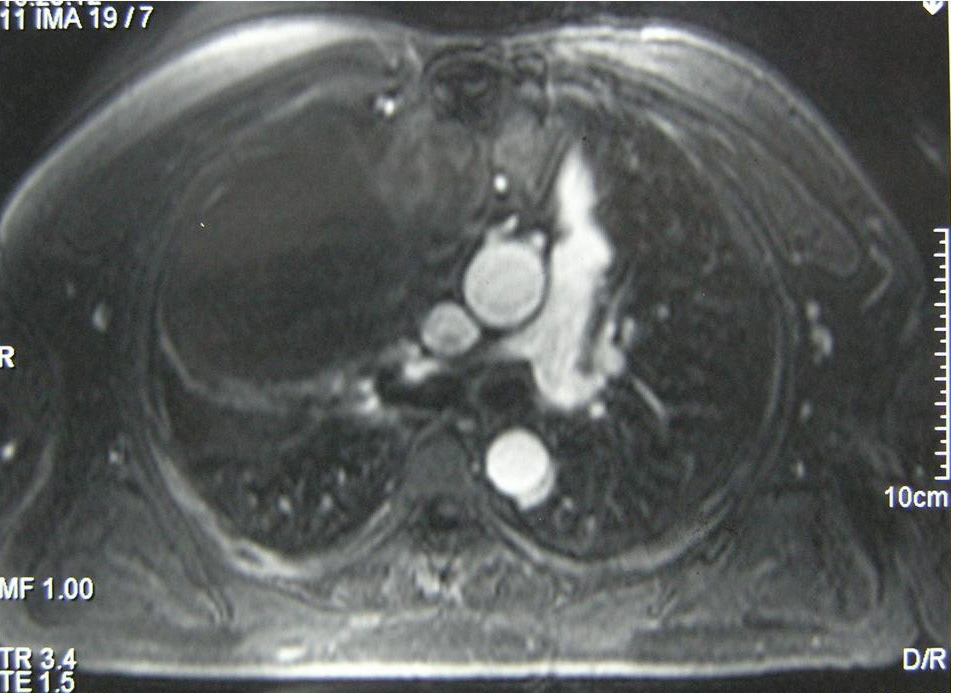
MRI transverse scan showing mass extension and anatomical relationship between the tumour and the tree grafts at level of ascending aorta.

Standard chest radiography followed by computed tomography (CT) scan and magnetic resonance imaging (MRI) showed a mass measuring 15 cm in diameter, located in the anterior mediastinum with extension to the right hemithorax (Figure 1, 2). MRI showed a contiguity between the mass and the bypass grafts without signs of invasion (Figure 1, 2). Electrocardiography (EKG) and exercise stress test were also negative for cardiac disorders a coronary angiography was however performed to confirm the patency and functioning of the bypass.

The patient, whose symptoms worsened, was submitted to surgery without further investigations since it was thought that a preoperative histological determination would not change the therapeutic approach.

The surgical approach was a re-sternotomy with opening of the right pleural cavity. Exploration revealed a mass abutting sternal periosteum from which it could be separated. The mass extended from the aortic arch into the anterior mediastinum and most of the right pleural cavity without invading the lung. The main surgical problem was to be able to preserve aorto-coronary bypass which consisted of three venous grafts from ascending aorta to the main coronary vessels. The mass was isolated starting from the aortic arch, a dissection plane was there found, making it possible to separate the venous grafts. Right pericardium was however infiltrated and was removed permitting radical excision of the mass. The postoperative course was uneventful and the patient was discharged on seventh postoperative day.

**Figure 3:**
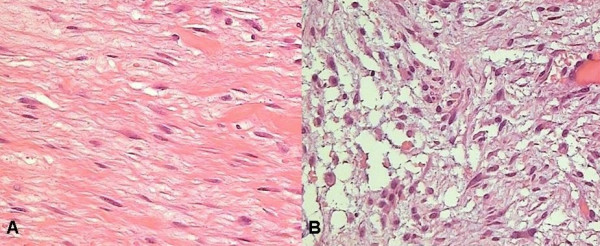
Photomicrograph showing the tumour. A. Fusiform cells organized in fasciae, B. Fascitis like mixoid degeneration (Haematoxylin and Eosin 400×)

The histological examination of the mass revealed a mesenchymal tumour with fibromixoid features, the ultrastructure of which was that of an aggressive fibromatosis (Figure 3). At a 3 years follow-up the patient is still recurrence free.

## Discussion

Literature search revealed only one more case of an intra-thoracic desmoid tumour in a patient with an aorto-coronary bypass, described by Kostonly *et al*., in 2001 [4]. Other cases of intra-thoracic tumours described in the literature are reported after a surgical trauma or after a thoracotomy [5,6], pulmonary lobectomy [7] and after silicone breast implants [8] with either involvement of the chest wall and an intra-thoracic growth.

The clinical picture of such tumours is not specific, meanly characterized by chest pain and/or dyspnoea due to compression of the airways or lung [5]. Radiological examination may be useful in indicating the presence of desmoid tumour, but diagnostic certainty and differential diagnosis with malignant tumours such as sarcoma can be achieved by histology. CT scans make it possible to define the location of the tumour, its behaviour with regard to contiguous organs as well as bone erosions [6-8]. At MRI, the behaviour of the tumour is heterogeneous related to fibrous tissue content, grade of cellularity and myxomatous degeneration which yields a signal of greater intensity in T2W1 when present to a greater extent [9,10], as in the case reported here.

Different therapeutic approaches have been proposed for thoracic desmoid tumours. Surgical treatment is currently indicated. Resection must be as radical as possible since the quality of the excision appears to have an impact on recurrence rates. Partial resection of a desmoid tumour has been reported but the residual tumour was present at one year follow-up [2,11] despite adjuvant chemotherapy [2]. Recurrences after radical surgery is anyway reported in up to 40% [12,13].

Cardoso *et al*., has reported radiotherapy combined with surgery in a patient where removal of the tumour required the sacrifice of the great vessels involved and their replacement with prostheses. In this case the patient was disease-free at a 6-year followup [14].

Surgical treatment of thoracic desmoid tumours have to take into account problems related to the resection of contiguous structures with their possible replacement either by autologus or prothesic graft with increased postoperative morbidity. In the case reported by Kostolny *et al*., the saphenous-vein coronary bypass, combined with anastomosis of the left internal mammary artery to the left anterior descending coronary artery, was infiltrated and the construction of a substitute saphenous-vein aortocoronary bypass proved therefore necessary [4]. In the case described here, the tumour could be separated from the bone and vascular structures of the mediastinum including the aorto-coronary bypass vessels, whereas the pericardium was resected, thus allowing a radical resection with no evidence of relapse at a three years follow up as a result.

## Conclusion

Although surgical treatment of intra-thoracic desmoid tumours may require extensive resection with possible reconstruction, immediate relief of increasing symptoms and follow up results make it actually the best therapeutic option.

## Competing interests

The author(s) declare that they have no competing interests.

## Authors' contributions

**GB **has conceived, coordinated other co-authors, draft and revised the manuscript. **AM **and **FG **have participated by acquisition and analysis of literature data and have helped to draft the manuscript. **GF **and **CC **have participated to conceive the manuscript and critically revised it for important intellectual content. **PI **has carried out the pathological findings.

All Authors read and approved the final manuscript.
